# Investigation of the surface mechanical properties of functionalized single-walled carbon nanotube (SWCNT) reinforced PDMS nanocomposites using nanoindentation analysis†

**DOI:** 10.1039/d4ra02717e

**Published:** 2024-05-10

**Authors:** Pavithra Ananthasubramanian, Rahul Sahay, Nagarajan Raghavan

**Affiliations:** a nano-Macro Reliability Laboratory, Engineering Product Development Pillar, Singapore University of Technology and Design 8 Somapah Road Singapore 487372 nagarajan@sutd.edu.sg

## Abstract

Functionalizing single-walled carbon nanotubes (SWCNT) with different chemical functional groups directly enhances their chemical adhesion and dispersion in viscous polymeric resins such as polydimethylsiloxane (PDMS). Nevertheless, the ideal surface polarity (hydrophilic or hydrophobic) for SWCNT to foster stronger chemical bonding with PDMS remains uncertain. This investigation delves into the impact of enhanced SWCNT dispersion within PDMS on the surface mechanical characteristics of this flexible composite system. We use carboxylic acid-functionalized SWCNT (COOH–SWCNT) and silane-functionalized SWCNT (sily–SWCNT), recognized for their hydrophilic and hydrophobic surface polarities, respectively, as reinforcing agents at ultra-low weight percentage loadings: 0.05 wt%, 0.5 wt%, and 1 wt%. We perform quasi-static nanoindentation analysis employing a Berkovich tip to probe the localized mechanical behavior of PDMS–SWCNT films at an indentation depth of 1 μm. Plastic deformation within the samples, denoted as plastic work (*W*_p_), as well as the elastic modulus (*E*), hardness (*H*), and contact stiffness (*S*_c_) of the composites are examined from the force–displacement curves to elucidate the enhancement in the surface mechanical attributes of the composite films.

## Abstract 2

Functionalizing single-walled carbon nanotubes (SWCNT) with different chemical functional groups directly enhances their chemical adhesion and dispersion in viscous polymeric resins such as polydimethylsiloxane (PDMS) improving the overall surface mechanical properties of the composite.

## Graphical abstract

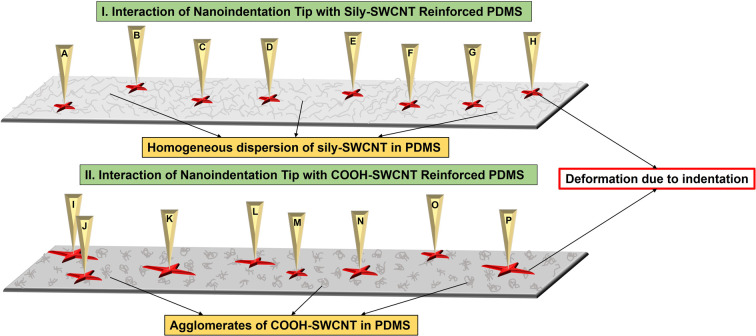

## 1 Introduction

Polydimethylsiloxane (PDMS) is one of the most extensively used silicone-based elastomers for modifying surfaces to control wettability, adhesion, and friction properties.^1,2^ Due to the inherent hydrophobic nature of PDMS, it is also one of the most used materials for hydrophobic coatings in surface engineering applications.^3,4^ As one of the most widely used elastomers in the field of cell mechanics, PDMS is subjected to several surface treatments such as plasma oxidation and UV sterilization, that directly affect the mechanical properties of PDMS.^5^ The minor changes imparted to the surface mechanical properties of PDMS are reported to cause significant errors in quantifying cellular traction forces.^6–8^

Since PDMS is a soft polymer with low elastic modulus in the range of 1–3 MPa in its unmodified conditions,^9–11^ attempts to improve its local mechanical properties using nanoscale reinforcements are prominent.^12,13^ Reinforcing PDMS with single-walled carbon nanotubes (SWCNT) is one of the most successful designs reported to improve the electro-mechanical properties of PDMS.^14–21^ However, SWCNT-reinforced PDMS composites are fabricated for limited applications attributed to the advanced infrastructural and process control requirements to produce defect-free SWCNT.^22^ In the last decade, PDMS–SWCNT-based composite systems have been produced for specific applications such as saturable absorbers,^18,19^ proton radiation shielding,^20^ wearable electronics,^23^ novel multilayered structures with improved fracture mechanics,^14^ porous electrodes,^21^ and selective permeation membranes of industrial gases.^24^ Homogeneous dispersion of carbon nanotubes (CNT) in a viscous PDMS matrix is a challenge that has been limiting the possibility of upscaling the manufacturing of this material system for multifunctional applications on a large scale.^25–28^ Interfacial characteristics such as filler dispersion, orientation, waviness, and adhesion between CNT and PDMS determine the final properties of the composite.^29^ Aggregation or agglomeration of CNT in PDMS leads to inhomogeneous dispersion that negatively impacts the properties of the composite. This occurs even at low weight percentage loadings due to the high surface energy of CNT.^30^

To overcome this, mechanical shearing or chemical functionalization of CNT has been widely explored to improve CNT dispersion in PDMS.^27,28,31–33^ While multiwalled carbon nanotubes (MWCNT) manage to retain their structural integrity upon the use of mechanical forces to disperse them in viscous polymeric matrices, SWCNT loses their structural traits such as length, and defect-free sp^2^ hybridization upon the use of extensive mechanical shear.^34^ Young's modulus values obtained for SWCNT^35^ and MWCNT^36^ from direct tensile loadings are in the ranges of 320–1470 GPa and 270–950 GPa respectively, indicating the superiority of SWCNT over MWCNT. Electrical conductivities of SWCNT and MWCNT have also been reported on the order of 10^2^ to 10^6^ S cm^−1^ and 10^3^ to 10^5^ S cm^−1^ respectively.^37^ Due to the better electrical and mechanical properties of SWCNT over MWCNT, SWCNT is preferred as a filler for PDMS for electromechanical devices that demand cutting-edge performance.^38^

To disperse SWCNT in PDMS without the use of mechanical dispersion techniques, chemical functionalization of SWCNT is reported as a successful method that facilitates a direct technique to disperse the low-dense SWCNT fillers in the viscous PDMS matrix.^39^ In previous reports, carboxylic acid-functionalized MWCNTs (COOH–MWCNT) and hydroxyl-terminated MWCNTs (OH–MWCNT) have been studied comparatively against unfunctionalized MWCNTs as fillers on PDMS for improvement in dispersion in PDMS and the resulting electrical,^40,41^ and thermal properties.^42^ Lu Bai *et al.*,^43^ comparatively studied COOH-functionalized CNT and OH-terminated polydimethylsiloxane functionalized CNT (OH–PDMS–CNT) as fillers on poly(methylphenylsiloxane) (PMPS) composites. With OH–PDMS–CNT as a filler, improvements in the interfacial adhesion, and thermal and macroscopic tensile mechanical properties are reported. The improvement is attributed to two reasons: (i) hydrogen bonds formed between OH–PDMS–CNT and PMPS, and (ii) the –OH groups in OH–PDMS react with PMPS forming an additional chemical crosslinking. Their study emphasizes the importance of chemical adhesion between the filler and matrix to effectively improve the desired composite properties. In another study by T. P. Chua *et al.*,^44^ diphenyl-carbinol (DPC) and silane (sily) functionalized MWCNTs are comparatively studied as fillers on PDMS. The effect of the better-dispersed composite on the improved dynamic mechanical properties and electrical and thermal conductivities are discussed. From all these reports, it is observed that CNTs with hydrophilic surface polarities (OH–CNT/COOH–CNT) and hydrophobic surface polarities (DPC–CNT/sily–CNT) have been interchangeably used on PDMS as a reinforcing filler. However, there is no report comparatively evaluating the role of surface polarity of the functionalized CNTs (hydrophilic *vs.* hydrophobic) on the interfacial chemical adhesion between CNT and PDMS. We hypothesize that SWCNT with a hydrophobic surface polarity achieved through a facile silane functionalization would impart better dispersion and interfacial adhesion of sily–SWCNT with PDMS. This shall be achieved jointly because of the compatible hydrophobic surface polarities of sily–SWCNT and PDMS and the formation of Si–O–Si covalent bond between the silane groups on SWCNT and siloxane groups in PDMS.^45^ In a previous study, our group reported an improvement in the dispersion of SWCNT in PDMS using a hydrophobic silane functionalization.^17^ The effect of the improved dispersion is reflected on the enhanced optical and macroscopic mechanical properties such as the optical transmittance, elastic modulus, bonding strength, and debonding time. The study focussed on estimating the three-dimensional macroscopic mechanical properties.

The degradation and failure of PDMS–SWCNT-based soft composite films begins to happen through the weakest links which are usually on the surface of a material. Surface defects act as nucleation sites for cracks, contributing significantly to failure initiation in thin films.^46–48^ Localized surface mechanical properties of PDMS-based soft materials reveal valuable information on the mechanical robustness of the fabricated microstructure by investigating the surface properties of the sample at a nanoscale.^49,50^ Classical methods of material testing become extremely difficult or almost impossible at the micro or nanoscale. It is also challenging to conduct conventional mechanical tests on soft materials, that have limited dimensions and complex microstructures. Depth sensing indentation is one of the testing methods that can be fine-tuned and tested on such samples at multiple length scales.^51^ Localized nanoindentation analysis is a reliable test to compare the homogeneity of the achieved mechanical properties at the nanoscale with macroscopic mechanical tests such as delamination and dynamic mechanical analysis.^52–55^

Nanoindentation analysis has been explored as a reliable technique by several research groups to estimate the surface properties and nanomechanics of soft materials such as biological tissues with heterogeneous microstructures and irregular dimensions.^56^ Localized hardness and modulus of biomaterials such as hydrogels^57–59^ and cartilage^60,61^ have been reported from nanoindentation analysis. Nanoindentation analysis of PDMS has also been reported by several research groups over the last two decades across several length scales and a range of sample thicknesses to understand the localized mechanics for different crosslinking methods/ratios, ages, and fluid environments.^10,12,49,50,55,62–69^ From a recent study by Arevalo, S. E. *et al.*,^70^ it is understood that the number of publications related to understanding the nanomechanics and surface properties of soft polymers has increased 3× in 2022 since the early 2000s. Table 1 briefly summarizes the nanoindentation analysis done by several research groups on Sylgard® PDMS using different analysis methods and the objectives behind each type of study. As indicated in the table, this study is one of the first studies to evaluate the surface mechanical properties of SWCNT-reinforced PDMS composites. The study evaluates the effect of the chemical surface functionality of SWCNT on the quality of reinforcement and imparted mechanics to PDMS. Nanomechanical properties evaluated from the surface of the composite such as the elastic modulus (*E*), hardness (*H*), and contact stiffness (*S*_c_) are discussed in detail. Quasi-static nanoindentation analysis used in this study employs a standard Berkovich tip under a displacement-controlled mode to locally probe the sample at a depth of 1 μm. The area under the loading–unloading curves reveals the mechanics behind the viscoelasticity of the sample.

**Table 1:** Literature review on the nanoindentation test conducted on Sylgard® 184 PDMS

S. no.	PDMS thickness (μm)	PDMS base: curing agent ratio	Curing temperature and time	Type of indentation tip	Mode of indentation	Purpose of study	Ref.
1	2500	10/15/20/25/30 : 1	RT/2 weeks	Cono-spherical diamond tip	Load-controlled	Estimation of elastic modulus as a function of PDMS crosslinking density	67
2	3000	5 : 1	65 °C/1 h	Berkovich & flat punch tips	Displacement-controlled	Estimation of compressive elastic modulus	49
3	2500	10/15/20/25/30 : 1	RT/2 weeks	Cono-spherical diamond tip	Load-controlled	Consideration of adhesion energy in elastic modulus	74
4	1000–2000	5/7/10/16.7/20/25/30/33 : 1	65 °C/24 h	Spherical/Berkovich/cube-corner/conical tips	Displacement-controlled	Estimation of elastic modulus as a function of PDMS crosslinking density and sample thickness	10
**5**	**500**	**10 : 1**	**70 °C/4 h**	**Berkovich tip**	**Displacement-controlled**	**Estimation of elastic modulus, hardness, and contact stiffness as a function of the surface functionality of SWCNT reinforced in PDMS**	**This work**

In this study, carboxylic acid functionalized SWCNT (COOH–SWCNT) and silane functionalized SWCNT (sily–SWCNT), respectively with hydrophilic and hydrophobic surface polarities, are comparatively studied as reinforcing fillers on PDMS at ultra-low loadings: 0.05 wt%, 0.5 wt%, and 1 wt%. The novelty of this study lies in three parts listed as follows:

(i) Since crosslinked PDMS in its unmodified condition is a polymer with one of the highest reported hydrophobic surface polarities,^71^ we choose hydrophobic (sily–SWCNT) and hydrophilic (COOH–SWCNT) SWCNTs to comparatively study the effect of opposite surface polarities on the homogeneity in reinforcement of PDMS and the resulting surface mechanical properties.^26,71–73^ This is one of the first studies to evaluate the effect of surface polarity of CNT achieved from chemical functionalization on the dispersion and interfacial adhesion between the filler and matrix.

(ii) This is also one of the first reports to evaluate the effect of improved filler dispersion and interfacial adhesion between functionalized SWCNT and PDMS on the surface mechanics of the composite. Enhancement in the local surface mechanical properties investigated at a nanoscale is a strong indicator of the homogeneity in the dispersion of SWCNT in PDMS at high resolution.

(iii) Nanoindentation analysis is chosen as a nano-scale probing method to indent the sample and evaluate the surface mechanical properties. Since the nanoindentation technique tests the sample locally at single individual points, this is one of the first studies to report the variability of surface mechanical properties across a sample at several points. Each sample is tested at over 50 points to assess the repeatability of the results across a sample. This is also one of the first studies on PDMS-based composites to evaluate the force–displacement curves obtained using nanoindentation analysis in detail to evaluate the specific surface mechanical properties of the test structures. The slope of the unloading curve for each point on every sample is investigated to calculate the elastic modulus (*E*), hardness (*H*), and contact stiffness (*S*_c_).

## 2 Experimental

### 2.1 Materials

PDMS (SYLGARD® 184) Elastomer Kit is purchased from Dow Corning, US. Carboxylic acid functionalized single-walled carbon nanotubes (COOH–SWCNT) (>90% carbon basis, with length 4–5 nm and diameter 0.5–1.5 μm, bundle dimensions), and 3-aminopropyltriethoxy silane, 99% (APTES) are purchased from Merck-Sigma Aldrich, Singapore.

### 2.2 Methods

#### 2.2.1 Fabrication of test structures

Silane functionalization of COOH–SWCNT is conducted using a previously reported procedure.^75,76^ COOH–SWCNT and sily–SWCNT are dispersed in the PDMS matrix using a facile solvent-mediated dispersion process. Solvent-mediated dispersion of SWCNT in PDMS and fabrication of composite films using a solution casting technique are conducted following the same technique reported by our group previously.^17^ The sample thickness is ∼500 μm.

#### 2.2.2 Characterization of test structures using nanoindentation analysis

Quasi-static nanoindentation analysis is conducted in a displacement-controlled mode using Hysitron TriboIndenter TI 950. A standard Berkovich tip is used to indent all the samples up to a depth of 1 μm. The loading and unloading rates of the tip during the test are maintained as 5 nm s^−1^ and 10 nm s^−1^ respectively for all samples. Each sample is tested at over 50 points to assess the repeatability of the surface mechanical properties of the test structures. Two consecutive points of test in each sample are separated by a minimum spatial distance of 40 μm to avoid Mullin's effect.^49,77,78^

## 3 Results and discussion

### 3.1 Structural analysis of test structures

**Fig. 1:**
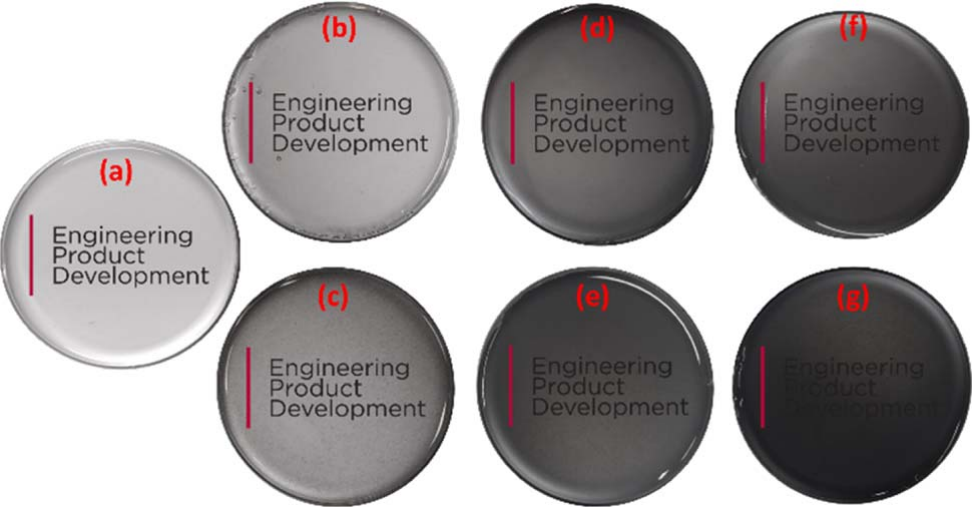
Photographic images of the fabricated test structures: (a) neat PDMS, (b) PDMS–SWCNT (sily, 0.05 wt%), (c) PDMS–SWCNT (COOH, 0.05 wt%), (d) PDMS–SWCNT (sily, 0.5 wt%), (e) PDMS–SWCNT (COOH, 0.5 wt%), (f) PDMS–SWCNT (sily, 1 wt%), (g) PDMS–SWCNT (COOH, 1 wt%).

The samples are structurally characterized using photographic and field emission scanning electron microscopic imaging (FESEM). Fig. 1 shows the photographic images of the fabricated test structures imaged against the department logo of ‘Engineering Product Development’ at the Singapore University of Technology and Design. The pictures qualitatively reveal the difference in the transparency of the fabricated test structures. Sily–SWCNT-loaded PDMS samples are more transparent than the respective COOH–SWCNT-loaded PDMS samples. As quantified in our previous study,^17^ the transparency of the sily–SWCNT reinforced PDMS is better over COOH–SWCNT reinforced PDMS by 1.2×, 1.8× and 1.1× respectively across 0.05 wt%, 0.5 wt% and 1 wt% SWCNT loadings. This difference is a clear implication of the better dispersion of sily–SWCNT in PDMS over COOH–SWCNT.

**Fig. 2:**
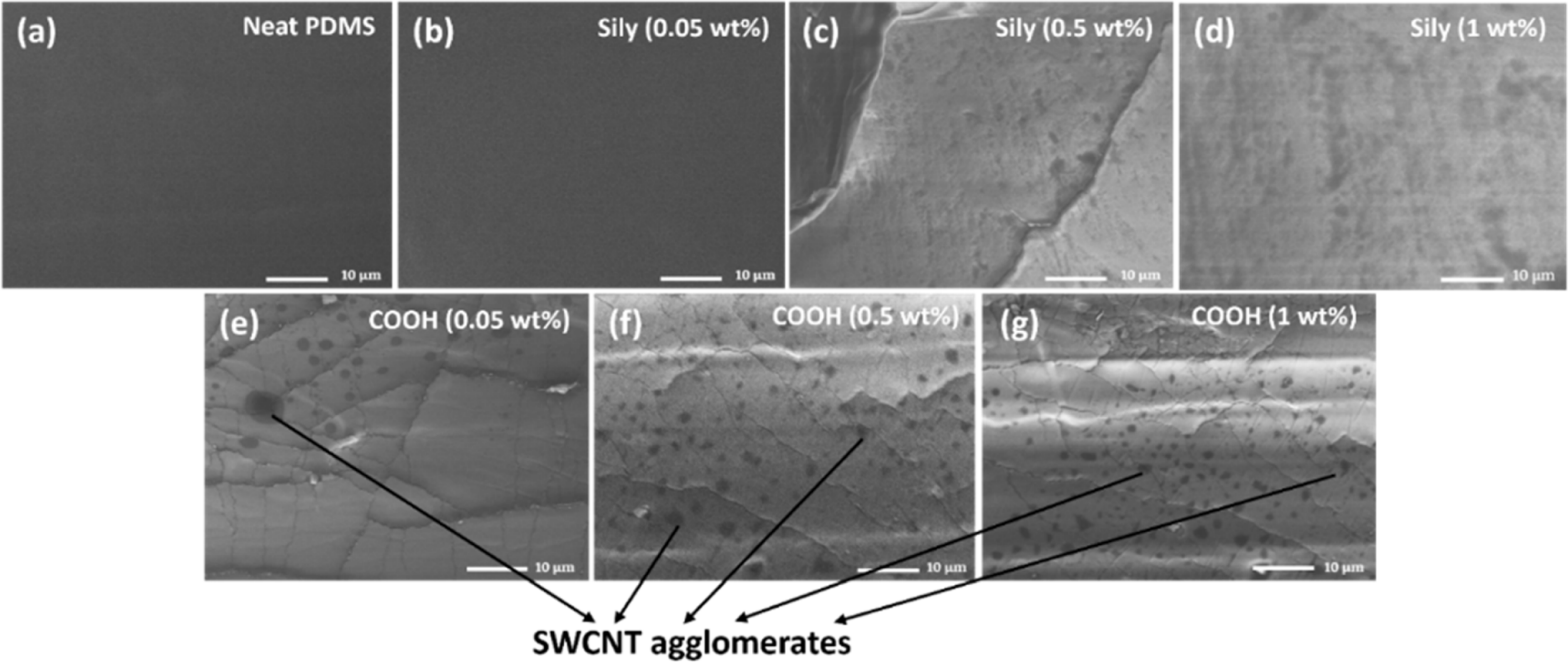
Cross-sectional FESEM analysis of the test structures: (a) neat PDMS, (b) PDMS–SWCNT (sily, 0.05 wt%), (c) PDMS–SWCNT (sily, 0.5 wt%), (d) PDMS–SWCNT (sily, 1 wt%) (e) PDMS–SWCNT (COOH, 0.05 wt%), (f) PDMS–SWCNT (COOH, 0.5 wt%), (g) PDMS–SWCNT (COOH, 1 wt%).

This can be further seconded using a cross-sectional FESEM analysis. Fig. 2 shows the FESEM images of the test structures. The COOH–SWCNT loaded composite samples (Fig. 2(e)–(g)) show distinct SWCNT agglomerates while the sily–SWCNT loaded composites show less prominent agglomerates (Fig. 2(b)–(d)) indicating the better dispersion of sily–SWCNT in PDMS matrix. FTIR analysis of the functionalized SWCNT (sily and COOH) and the composite films are conducted, and the results are discussed in Section S1 of the ESI.† From the analysis, it is understood that the silane functionalization of SWCNT creates a chemical Si–O–Si adhesion between sily–SWCNT and PDMS.^17^

Contact angle measurements of functionalized SWCNT (COOH and sily) are comparatively conducted to ensure the respective hydrophilic and hydrophobic surface polarities imparted to the nanotubes from the chemical functionalization. The results reveal that COOH–SWCNT is highly hydrophilic with the contact angle values dropping to less than 10° within 1 s from the contact of the water droplet onto the surface of COOH–SWCNT coated on a glass slide. However, in the case of sily–SWCNT, the contact angle value is stable at ∼140° for over 30 s from the initial contact time of the water droplet onto the surface of sily–SWCNT coated on a glass slide. This emphasizes the highly hydrophobic nature of the sily–SWCNT. The results from contact angle measurements are thoroughly discussed in Section S2 of the ESI.†

### 3.2 Nanoindentation analysis of test structures

**Fig. 3:**
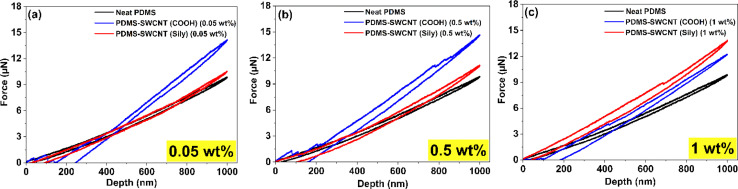
Loading–unloading plots from nanoindentation analysis of the test structures at different SWCNT loadings on PDMS: (a) 0.05 wt%, (b) 0.5 wt% and (c) 1 wt%. The curves indicate the resistance shown by the samples to indentation force and the area under the curves indicate the plastic deformation undergone by the samples.

Nanoindentation analysis is conducted on all the test structures following the procedure discussed in Section 2.2.2. Fig. 3(a)–(c) show the loading–unloading plots respectively for 0.05 wt%, 0.5 wt%, and 1 wt% SWCNT loading on PDMS. Due to the very low concentrations of SWCNT in PDMS, the resistance to nanoindentation force values is not significantly different across any of the samples. Interestingly, PDMS–SWCNT (COOH) samples show marginally higher resistance over sily–SWCNT–PDMS samples at 0.05 and 0.5 wt%. The loading–unloading plots of each of the samples is a representative curve extracted from the extensive nanoindentation analysis that was conducted at over 50 individual points on each sample. The representative curves shown in these plots are the 50^th^ percentile values in terms of the resistance to the nanoindentation force exhibited by the samples. Hysteresis loss in these nanoindentation plots refers to the energy dissipated during the loading and unloading cycles of an indenter as it penetrates and retracts from a material's surface.^62,79^ This energy loss is typically represented by the area between the loading and unloading curves on a nanoindentation plot. In this study, it is referred to as plastic work (*W*_p_) as the area under the curve is a representation of the irreversible plastic deformation that the material undergoes. As represented in eqn (1), *W*_p_ is the difference between the total work (*W*_t_) (area under the loading curve) and the elastic work (*W*_e_) (area under the unloading curve).

1 *W*_p_ = *W*_t_ − *W*_e_

**Fig. 4:**
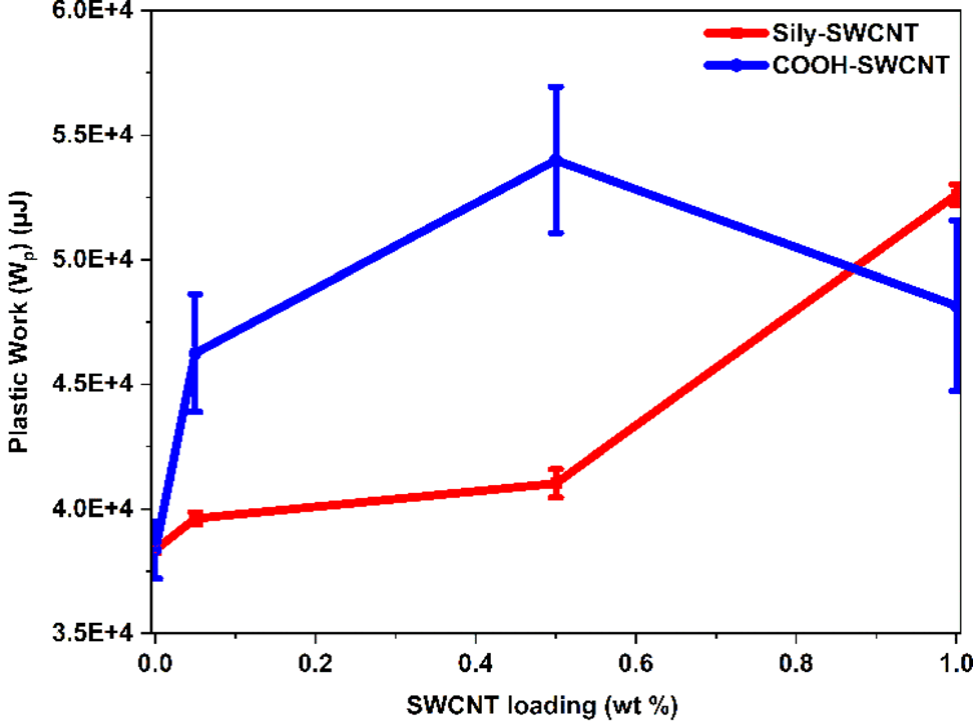
Plastic work (*W*_p_) undergone by PDMS–SWCNT composite samples during nanoindentation analysis.

PDMS is a viscoelastic material that retracts to its original dimensions upon the removal of an applied force. The incorporation of mechanically robust SWCNT in PDMS increases the plasticity of the elastic PDMS material and therefore enhances the overall viscoelastic mechanics.^80–82^Fig. 4 is a plot of the average area under the curves indicating the *W*_p_ undergone by each of the samples. The plot represents the average of three *W*_p_ values of each sample along with the respective standard deviation. As expected, the area under the curve for PDMS–SWCNT polymer nanocomposite tends to increase with an increase in the amounts of SWCNT, even at very low SWCNT loadings.

While PDMS–SWCNT (sily) samples show an increasing trend in *W*_p_ with increasing sily–SWCNT loadings, it is interesting to note an increasing trend followed by a decreasing trend in the case of PDMS–SWCNT (COOH) samples. The increase followed by a decreasing trend in the *W*_p_ for COOH–SWCNT loaded PDMS samples at 1 wt% SWCNT loading can be attributed to the increased agglomeration of COOH–SWCNT in PDMS. As the dispersion and chemical adhesion of COOH–SWCNT with PDMS is not as good as that of sily–SWCNT (seconded using physical photos, FESEM analysis, and FTIR analysis in Section 3.1), COOH–SWCNT tends to form agglomerates in the PDMS matrix. This effectively reduces the homogeneity of COOH–SWCNT distribution on the surface. Agglomeration of COOH–SWCNT facilitates the nanoindentation tip to encounter larger chunks of COOH–SWCNT in some areas and neat PDMS in other areas as the reinforcement is not homogeneous. This leads to a lowering of the overall *W*_p_ of the PDMS–SWCNT (COOH) at a higher weight percentage COOH–SWCNT loading in PDMS.

### 3.3 Estimation of reduced elastic modulus (*E*_r_) and elastic modulus (*E*) from nanoindentation analysis

This section focuses on the estimation of the reduced elastic modulus (*E*_r_) and elastic modulus (*E*) of SWCNT-reinforced PDMS from the nanoindentation plots. The load–displacement plots were analyzed using the Oliver–Pharr method, a well-established approach for determining mechanical properties from sharp indentation data.^83^ The contact area (*A*_c_) between the Berkovich indenter and the samples was calculated using the following empirical relationship:2
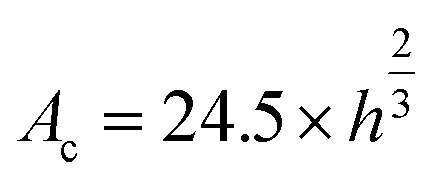
where *h* represents the penetration depth in nm.^84^

**Fig. 5:**
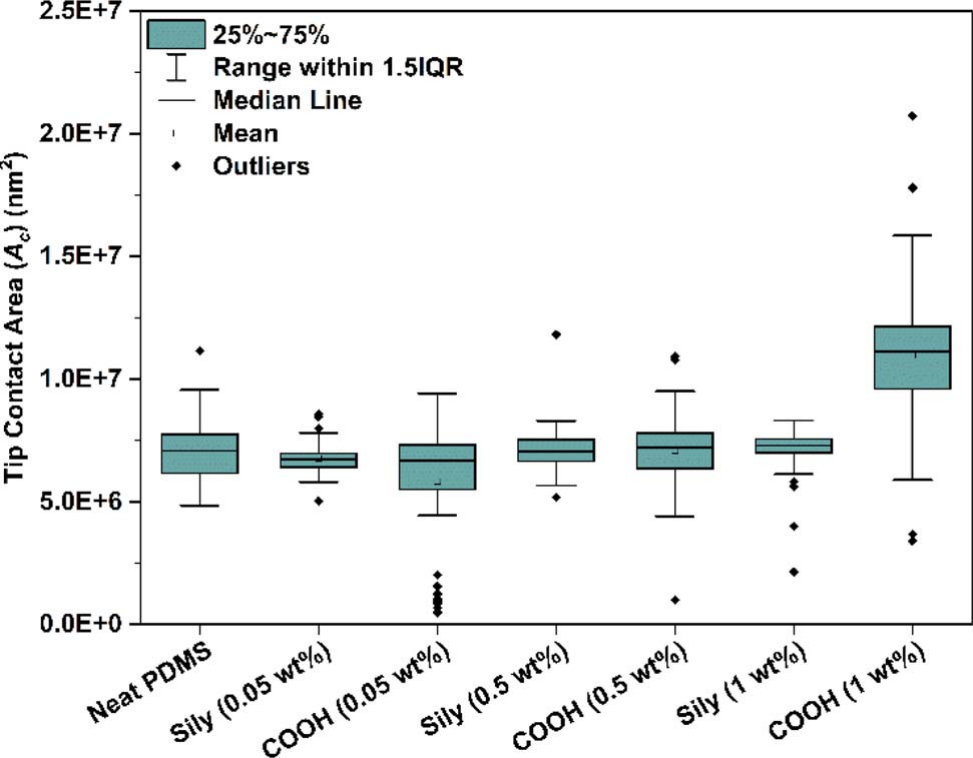
Box plot of the contact area (*A*_c_) of the tip with all analysis points on each sample.

A box plot of the *A*_c_ of the tip across all points of analysis in a sample is depicted in Fig. 5. It can be observed from the box plot that the contact area of the tip is not consistent across all the samples though the depth of indentation (*h*) was constantly set to 1000 nm. Due to the inhomogeneity in the distribution of COOH–SWCNT in PDMS, the variance in the *A*_c_ is significantly high in neat PDMS and COOH–SWCNT samples. This can be attributed to the viscoelastic nature of PDMS. PDMS exhibits both viscous (flow-like) and elastic (spring-like) behaviors. The addition of SWCNT affects both the elastic and viscous nature of PDMS individually in the following ways:

(i) Elasticity (spring-like nature): by adding SWCNT to PDMS, the material is reinforced which often increases the elastic modulus (discussed in detail in the coming sections).

(ii) Viscosity (flow-like nature): viscosity is the ability of a fluid to flow or deform over time when subjected to an applied force or stress. This behavior is a result of the polymer chains sliding past each other. Upon addition of sily–SWCNT in PDMS which chemically bonded with PDMS chains, it restricts the flow of PDMS chains under the applied load, adding to the stiffness of the PDMS against the applied load.

Due to the better reinforcement of sily–SWCNT in PDMS, the samples offer a stiffer surface for the tip to establish a consistent contact area for the tip, unlike PDMS–SWCNT (COOH) samples. In line with this, the variance in the *A*_c_ values for PDMS–SWCNT (sily) is reducing with an increase in CNT concentration.

The *E*_r_ was also determined using the Oliver–Pharr method:3
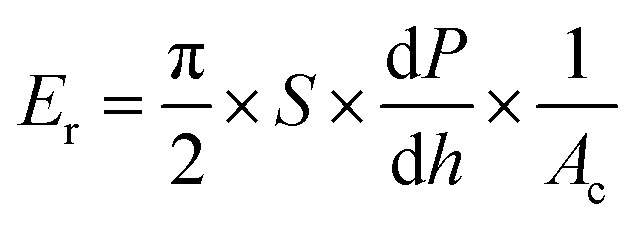
where *S* is the stiffness of the system, and d*P*/d*h* is the slope of the unloading curve. This method provides a reliable means of estimating *E*_r_ for elastic materials under nanoindentation conditions.^83^ Considering the nearly incompressible nature of PDMS, with a Poisson's ratio (*θ*) close to 0.5, the elastic modulus (*E*) is estimated using the following equation.^85^4
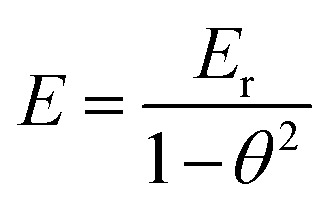


**Fig. 6:**
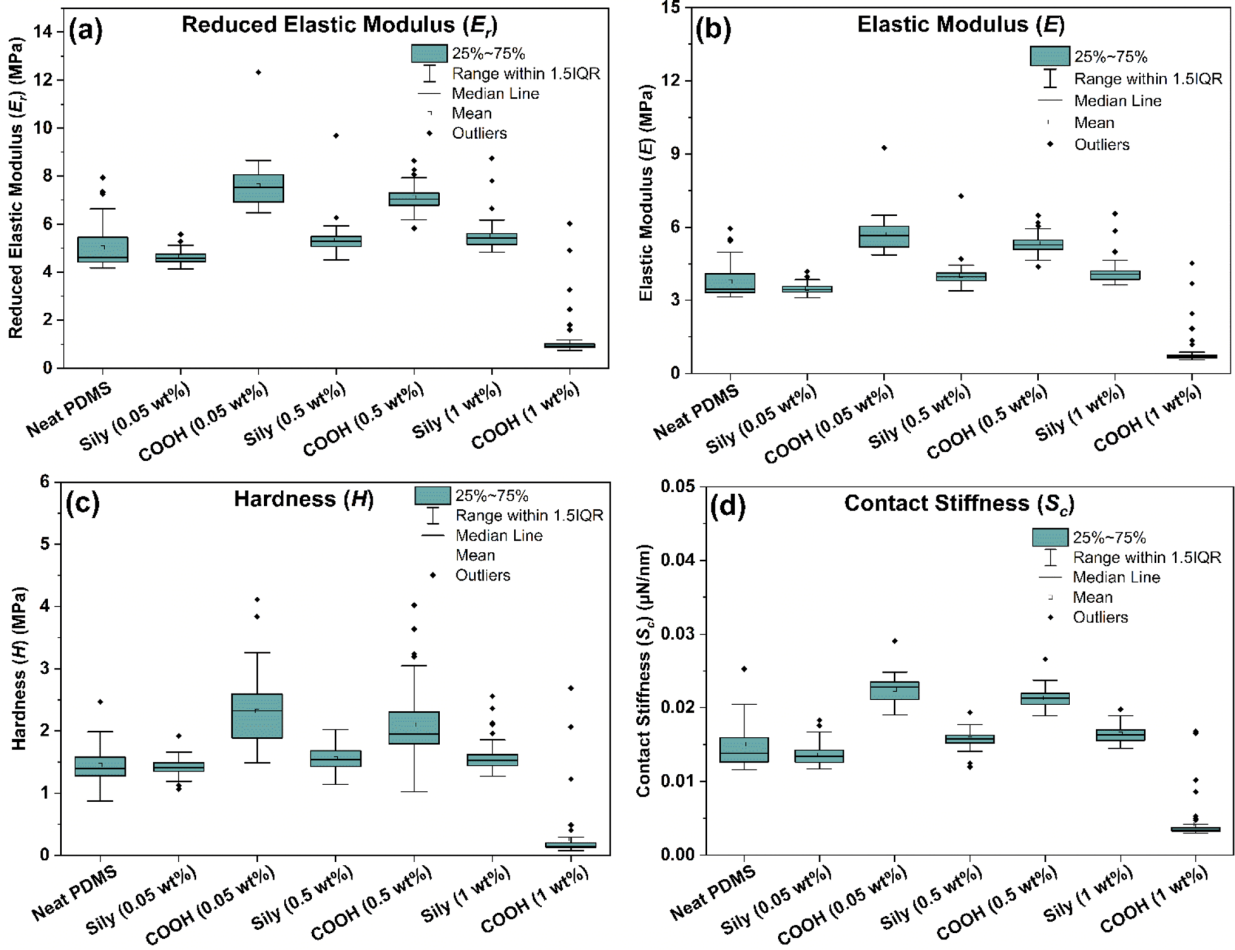
Box plots of (a) reduced elastic modulus (*E*_r_), (b) elastic modulus (*E*), (c) hardness (*H*) and (d) contact stiffness (*S*_c_) estimated through nanoindentation analysis.

Fig. 6(a) and (b) are box plots of *E*_r_ and *E* calculated from the slope of the unloading curves in Fig. 3 for all the 50 points of analysis on the samples using eqn (3) and (4). From the box plots, it can be understood that the outliers and variance values are higher in COOH–SWCNT reinforced PDMS across 0.05, 0.5, and 1 wt% samples indicating the inhomogeneity in the reinforcement. As the chemical adhesion and dispersion of sily–SWCNT has improved in PDMS, the outliers and variance of the moduli values are much lesser. The mean, median, lower quartile value, upper quartile value, and variance (highlighted in italic font) are summarized from the box plots in Table 2. It can be understood that the variance values for *E*_r_ and *E* are quantitatively lesser with improved surface mechanics for sily–SWCNT reinforced PDMS.

**Table 2:** Summary from the box plots of (a) reduced elastic modulus (*E*_r_), (b) elastic modulus (*E*), (c) hardness (*H*), and (d) contact stiffness (*S*_c_)

S. no.	Sample name	Median	Mean	25% lower quartile value	75% upper quartile value	Variance
**(A) Reduced elastic modulus (*E***_**r**_ **) (MPa)**						
1	Neat PDMS	4.59	5.02	4.43	5.45	*1.02*
2	Sily (0.05 wt%)	4.58	4.64	4.44	4.76	*0.32*
3	COOH (0.05 wt%)	7.53	7.6	6.91	8.05	*1.14*
4	Sily (0.5 wt%)	5.28	5.35	5.07	5.50	*0.43*
5	COOH (0.5 wt%)	7.04	7.12	6.78	7.29	*0.51*
6	Sily (1 wt%)	5.43	5.52	5.15	5.61	*0.46*
7	COOH (1 wt%)	0.91	0.07	0.87	1.01	*0.14*

**(B) Elastic modulus (*E*) (MPa)**						
1	Neat PDMS	3.47	3.77	3.31	4.10	*0.79*
2	Sily (0.05 wt%)	3.44	3.48	3.33	3.57	*0.24*
3	COOH (0.05 wt%)	5.65	5.71	5.18	6.04	*0.86*
4	Sily (0.5 wt%)	3.96	4.02	3.80	4.12	*0.32*
5	COOH (0.5 wt%)	5.28	5.34	5.09	5.47	*0.38*
6	Sily (1 wt%)	4.07	4.15	3.86	4.21	*0.35*
7	COOH (1 wt%)	0.69	0.05	0.65	0.76	*0.11*

**(C) Hardness (*H*) (MPa)**						
1	Neat PDMS	1.4	1.45	1.28	1.58	*0.30*
2	Sily (0.05 wt%)	1.41	1.42	1.35	1.49	*0.14*
3	COOH (0.05 wt%)	2.32	2.32	1.89	2.59	*0.70*
4	Sily (0.5 wt%)	1.54	1.56	1.43	1.68	*0.25*
5	COOH (0.5 wt%)	1.95	2.1	1.79	2.30	*0.51*
6	Sily (1 wt%)	1.53	1.58	1.44	1.62	*0.18*
7	COOH (1 wt%)	0.14	0.26	0.13	0.20	*0.07*

**(D) Contact stiffness (*S***_**c**_ **) (μN nm** ^**−1**^ **)**						
1	Neat PDMS	0.014	0.015	0.013	0.016	*0.003*
2	Sily (0.05 wt%)	0.014	0.014	0.013	0.014	*0.001*
3	COOH (0.05 wt%)	0.023	0.023	0.021	0.024	*0.003*
4	Sily (0.5 wt%)	0.016	0.016	0.015	0.016	*0.001*
5	COOH (0.5 wt%)	0.021	0.021	0.020	0.022	*0.002*
6	Sily (1 wt%)	0.016	0.016	0.016	0.017	*0.001*
7	COOH (1 wt%)	0.003	0.004	0.003	0.004	*0.001*

### 3.4 Estimation of hardness (*H*) and contact stiffness (*S*_c_) from nanoindentation analysis

The hardness (*H*) of a sample tested using nanoindentation analysis is calculated using the following equation.5
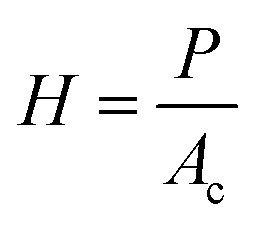
where *P* is the applied load and *A*_c_ is the contact area of the tip. Analysing hardness distribution across different regions of the sample reveals spatial variations in the material's mechanical properties. Fig. 6(c) is a box plot of the hardness of the samples tested at over 50 individual points on the sample. In line with the trend from the elastic moduli (*E*) data, the results from hardness (*H*) data reveal that in addition to improvement in the hardness value of PDMS–SWCNT (sily) samples at very low SWCNT loadings, the variance of the hardness values across the sample is less indicating a robust and homogeneous reinforcement imparted to PDMS by sily–SWCNT. Table 2 summarizes the median, mean, lower quartile and upper quartile values from the box plot in Fig. 6(c). The variance values are also calculated and listed and are highlighted in italic font. It is interesting to note that the variance in sily–SWCNT tends to reduce with increasing concentration of SWCNT in PDMS indicating a consistent dispersion.

The contact stiffness (*S*_c_) is indicative of a material's ability to resist deformation under load.^86^ This information is crucial for predicting the structural integrity and long-term performance of PDMS–SWCNT composites in real-world applications. Understanding the *S*_c_ helps in designing PDMS–CNT composites with specific mechanical properties, optimizing them for various applications such as sensors, actuators, or biomedical devices. In applications where PDMS–SWCNT composites are used as sensors or actuators, the contact stiffness affects the sensitivity and responsiveness of the material.^86–88^*S*_c_ is estimated by fitting a regression function to the upper part of the unloading curve. It is correlated using the following equation.

6 *S*_c_ = 2*βA*_c_π*E*_r_

where *β* is a constant that depends on the geometry of the indenter (*β* = 1.034 for a Berkovich indenter).^89^Fig. 6(d) shows the box plot of *S*_c_ of the samples estimated from nanoindentation analysis. The values, once again, emphasize the homogeneity of reinforcement imparted to PDMS by sily–SWCNT at a localized probing at a depth of 1 μm. The variance values are calculated and highlighted in italic font in Table 2.

**Fig. 7:**
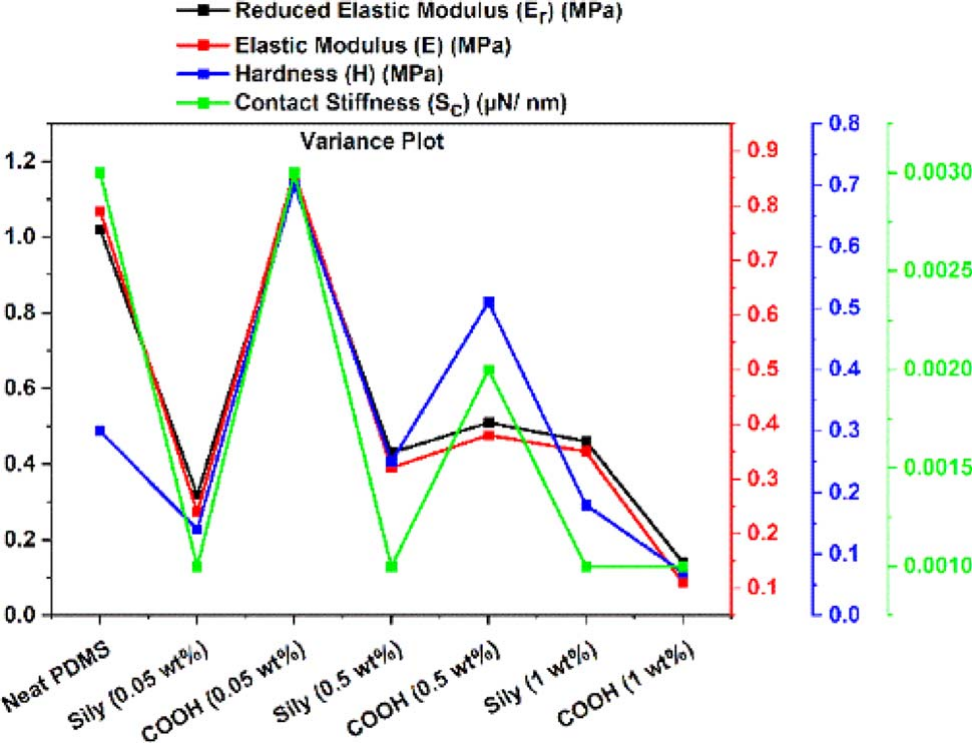
Variance in the values of reduced elastic modulus (*E*_r_), elastic modulus (*E*), hardness (*H*), and contact stiffness (*S*_c_) were tested at over 50 points on each sample.

Based on the box plots in Fig. 6 and the variance values listed in Table 2, a variance plot is plotted in Fig. 7 to better understand the trend. It is interesting to note that across all the four surface mechanical properties discussed in this study, sily–SWCNT reinforced PDMS has lesser variance with respect to its respective COOH–SWCNT reinforced PDMS counterpart. This indicates the homogeneous dispersion of sily–SWCNT in PDMS up to a precision of 1 μm depth. The viscous nature of neat PDMS leads to an inconsistent stiffness across its surface. Reinforcing PDMS with sily–SWCNT has improved the homogeneity of SWCNT dispersion in PDMS and the localized stiffness of the composite. The viscoelasticity of sily–SWCNT reinforced PDMS has improved leading to lesser variance in the properties across different points in a sample.

**Fig. 8:**
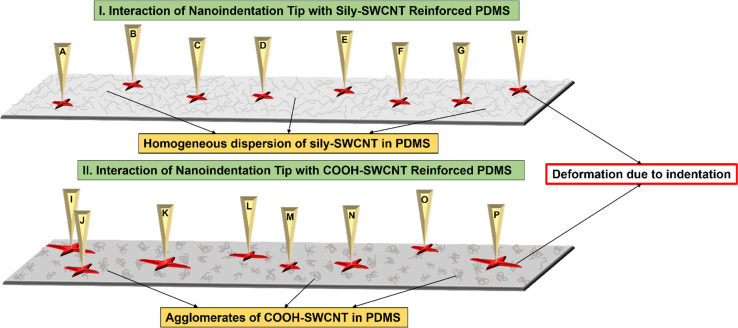
Schematic of the interaction of nanoindentation tip with (I) sily–SWCNT reinforced PDMS, and (II) COOH–SWCNT reinforced PDMS.

Based on these results, a schematic of the interaction of the nanoindentation tip with the PDMS–SWCNT composite samples is proposed in Fig. 8. Points A–H respectively represent random points of the nanoindentation test conducted on sily–SWCNT reinforced PDMS and points I–P respectively represent random points of the nanoindentation test conducted on COOH–SWCNT reinforced PDMS. Since the dispersion and chemical adhesion of sily–SWCNT are homogeneous in PDMS, the resistance offered by PDMS–SWCNT (sily) composite to the nanoindentation tip is uniform throughout the sample up to a precision of 1 μm depth.

As the hydrophilic COOH–SWCNT did not chemically adhere to the hydrophobic PDMS matrix, COOH–SWCNT failed to homogeneously disperse in the PDMS matrix. This leads to the formation of COOH–SWCNT agglomerates in the PDMS matrix. In this system, the distribution of COOH–SWCNT in PDMS is inhomogeneous. This leads the nanoindentation tip to interact with agglomerates of COOH–SWCNT (more elastic) at some points and neat PDMS (more viscous) at other points. Due to this, the resistance offered by the composite to nanoindentation tip is inconsistent across the sample leading to a large variance and outliers in the measurements made across the sample at over 50 tests.

Table 3 is a literature review of the nanoindentation analysis conducted on CNT-reinforced PDMS samples. The table collates the information reported in previous studies and compares the loading–unloading plots obtained from the nanoindentation analyses using a Berkovich tip to understand the surface mechanical properties of PDMS–CNT-based composite structures. None of the previously reported studies have dealt with the analysis of nanoindentation plots of PDMS–CNT composites in detail. The results majorly discuss the improvement in the resistance to indentation force because of the incorporation of CNT in the matrix and the associated *W*_p_ in the plots.

**Table 3:** Literature review on the nanoindentation test conducted on CNT reinforced PDMS composite films using Berkovich tip

S. no.	CNT wt% loading	PDMS thickness (μm)	PDMS base : curing agent ratio	Curing temperature and time	Depth of indentation (nm)	Maximum load (μN)	Plastic work (*W*_p_)	Ref.
1	0	3000	5 : 1	65 °C/1 h	5000 nm	80	191 260	49
2	0	45	15 : 1	80 °C/8 h	5000 nm	57	163 811	90
3	0	200	10 : 1	150 °C/15 min	1000 nm	13	8576	91
4	3				1000 nm	20	11 900	
**5**	**0**	**500**	**10 : 1**	**70 °C/4 h**	**1000 nm**	**10**	**38 355**	**This work**
**6**	**0.05-sily**					**11**	**39 624**	
**7**	**0.05-COOH**					**14**	**46 256**	
**8**	**0.5-sily**					**11**	**41 033**	
**9**	**0.5-COOH**					**15**	**53 994**	
**10**	**1-sily**					**14**	**52 612**	
**11**	**1-COOH**					**12**	**48 153**	

This is one of the first studies to report the surface mechanical properties of the soft PDMS–SWCNT composite in detail with specific emphasis on elastic modulus (*E*), hardness (*H*), and contact stiffness (*S*_c_). In addition, our study also emphasizes the statistical reliability of the measurements across different points on each sample. The results indicate that due to the surface functionalization of SWCNT and an efficient solvent-medicated dispersion process, the *W*_p_ values of the composites fabricated in this study are higher than the previously reported values.

It is convincing to note that the resistance to indentation force obtained in this study is comparable to the previous reports involving the same depth of indentation (1 μm). From the table, it is also understood that there are certain specific testing parameters during nanoindentation analysis that affect the output directly: (1) depth of indentation, (2) rate of loading and unloading of the tip, (3) thickness of the sample, and (4) sample formulation such as elastomer: curing agent ratio, curing cycle and fabrication process.

## 4 Conclusions

This study comparatively investigated the effect of surface functionalization on SWCNT on the surface mechanical properties imparted to PDMS composite films. The surface mechanical properties such as the resistance to nanoindentation force, plastic work (*W*_p_), elastic modulus (*E*), hardness (*H*), and contact stiffness (*S*_c_) are evaluated in detail. Hydrophobic silane functionalization facilitated an improved chemical adhesion and dispersion of sily–SWCNT in PDMS. The homogeneity in the dispersion of sily–SWCNT is commendable as the surface mechanical properties probed at a depth of 1 μm (0.2% of the total thickness of the sample) are in line with the macroscopic mechanical properties reported previously.^17^ The results provided in this study are tests conducted at over 50 different points on each sample. Each loading–unloading curve is individually analysed to calculate the elastic modulus (*E*), hardness (*H*), and contact stiffness (*S*_c_). The statistical reliability of the measurements is also investigated in detail. The variance in the data from sily–SWCNT reinforced PDMS is negligible. This is a testament to the homogeneity in the dispersion of sily–SWCNT in PDMS to the precision of 1 μm. Improvement in surface mechanical properties such as contact stiffness (*S*_c_) is crucial for the reliable sensitivity of PDMS–CNT-based wearable sensors.^86–88^ In addition, PDMS–CNT-based lab-on-a-chip devices and flexible microfluidic systems rely greatly on the elastic modulus and viscoelastic mechanics of the material system.^92–96^

In the field of mechanobiology, surface mechanical forces play a key role in maintaining tissue functions of the cells developed on PDMS substrates during and after the developmental stage.^5^ As a result, the impairment of mechanical forces on the substrate can lead to various diseases such as cardiac hypertrophy,^97,98^ arthritis,^99–101^ asthma,^102^ osteoporosis,^103^ deafness,^104,105^ atherosclerosis,^106^ cancer,^107^ glaucoma,^108^ and muscular dystrophy.^109^ To quantify the surface and intracellular forces involved during tissue growth, PDMS, attributing to its tuneable mechanical properties and high refractive index, is one of the most used substrate materials to mark force fields in traction force microscopy (TFM) and microfabricated post array detectors (mPADs) in mechanobiology laboratories.^5^ With such high significance of the surface mechanics of PDMS in several biomedical applications, our test structures with homogeneously improved surface mechanical properties and a standard method to reliably quantify the variability in surface mechanical properties using nanoindentation analysis hold great potential in mechanobiology labs.

In addition to wearable devices and mechanobiology, the bulk photovoltaic effect (BPE) in 1D nanotubes fabricated from carbon nanomaterials and transition metal dichalcogenides (TMD) is reported to be commendable.^110^ Recent reports evaluate the effect of strain-induced efficiency improvement in the BPE by an order of 1.9× in 1D nanotubes made from TMD supported on flexible polyethylene terephthalate as a substrate.^111,112^ This enhances the potential application of SWCNT-reinforced PDMS both as a mechanically robust substrate material for photovoltaic applications and as an active flexible material for energy harvesting. While SWCNT can be used as an active photovoltaic material, SWCNT-reinforced PDMS with improved surface and bulk mechanics can be used for strain-induced enhanced photovoltaic applications after optimizing the concentration of SWCNT in flexible PDMS to achieve adequate photovoltaic response.

In this study, hydrophobic silane functionalization is improving the anchorage between SWCNT and PDMS promoting load transfer from the soft PDMS to the mechanically robust SWCNT. This enhances the overall mechanical endurance of the soft composite system. Future work in this direction involves the probing of the composite system at different depths using micro and nano scratch techniques to estimate the coefficient of friction and advanced fracture mechanics. *In situ* FESEM assisted nanoindentation experiments also hold high potential in revealing the fracture mechanics involved during the deformation of PDMS-based soft composite materials. PDMS–SWCNT composites with improved surface and macroscopic mechanical properties find extensive electromechanical applications in wearable devices, strain sensors, and electromagnetic interference shielding materials.

## Author contributions

Pavithra Ananthasubramanian: conceptualization, methodology, validation, formal analysis, investigation, data curation, visualization, writing-original draft, writing-review and editing; Rahul Sahay: conceptualization, writing-review and editing, supervision; and Nagarajan Raghavan: conceptualization, writing-review and editing, supervision, project administration, funding acquisition.

## Conflicts of interest

There are no conflicts to declare.

## Supplementary Material

RA-014-D4RA02717E-s001
