# Supplementary material for: Investigation of the surface mechanical properties of functionalized single-walled carbon nanotube (SWCNT) reinforced PDMS nanocomposites using nanoindentation analysis
Source: RSC Adv. 2024 May 10;14(22):15249–60. doi: 10.1039/d4ra02717e (PMC11082875; doi:10.1039/d4ra02717e)
Supplement: RA-014-D4RA02717E-s001 [file RA-014-D4RA02717E-s001.pdf]

Electronic Supplementary Material (ESI) for RSC Advances.
This journal is © The Royal Society of Chemistry 2024

Investigation of the Surface Mechanical Properties of Functionalized Single-Walled Carbon Nanotubes (SWCNT) Reinforced PDMS Using

Supplementary Information

Nanoindentation Analysis

S1. FTIR Analysis

FTIR analysis of COOH-SWCNT and Sily-SWCNT are represented in Fig. S1 (a). ATR-IR analysis of neat PDMS and PDMS-SWCNT composite thin films are represented in Fig. S1 (b).

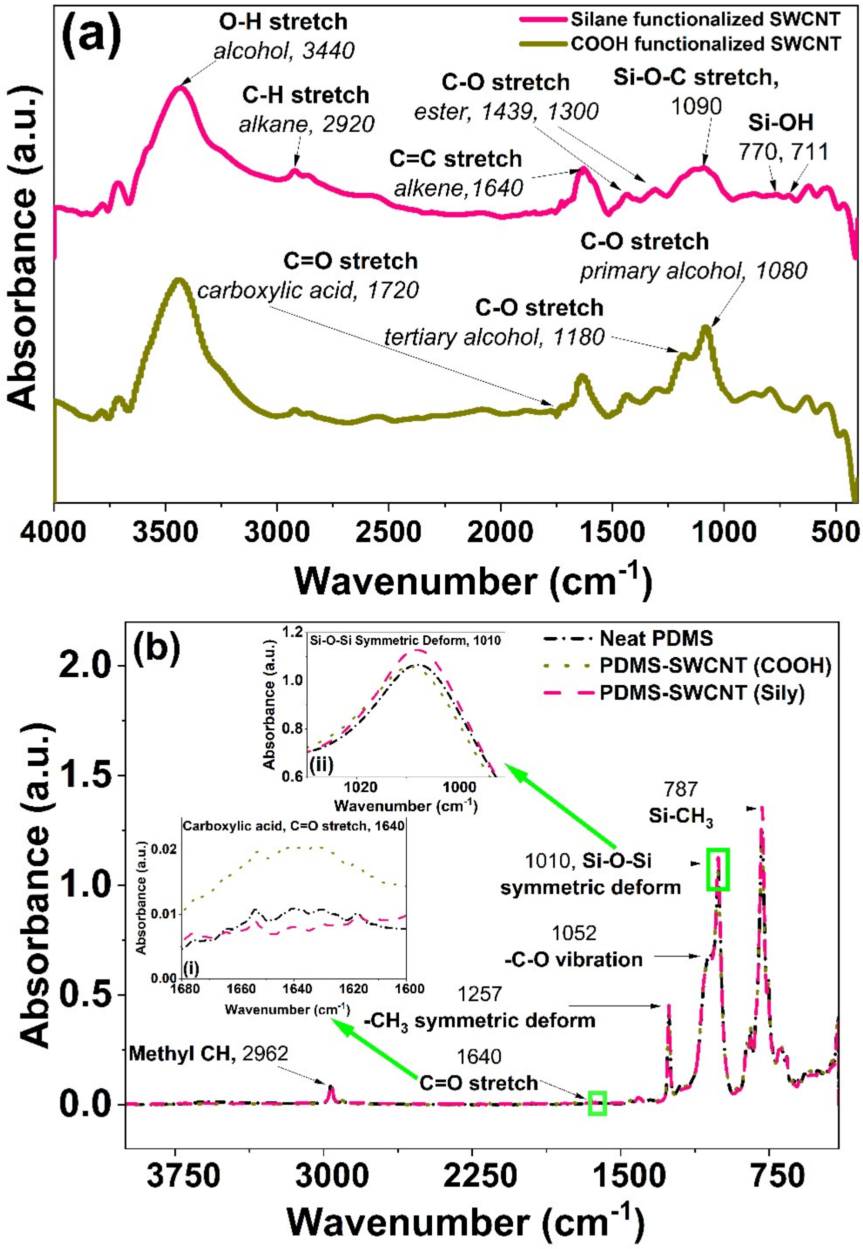

Figure S1. (a) FTIR analysis of COOH-SWCNT and Sily-SWCNT (b) ATR-IR analysis of neat PDMS, PDMS-SWCNT (COOH) and PDMS-SWCNT (Sily) thin films: inset (i) COOH peak from COOH-

SWCNT reinforced PDMS, inset (ii) mild difference in intensities of Si-O-Si peak across all thin films.

Fig. S1 (a) shows the identified signature vibrations for COOH functionalization and silane functionalization on SWCNT. In COOH-functionalized SWCNT, vibrations at 1720 cm^{-1} and 1064 cm^{-1} correspond to C=O and C-O stretches of the -COOH functional group. The absorption peaks at 3425 cm^{-1} indicate the presence of hydroxyl groups (-OH) in the carboxylic group [1].In the FTIR plot of Sily-SWCNT, the peak at 1141 cm^{-1} confirms the formation of a covalent chemical bond between silane and SWCNT (Si-O-C vibration). Weak signals at 757 cm^{-1} and 701 cm^{-1} represent Si-OH bond vibrations. Notably, the appearance of a weak signal at 1338 cm^{-1} corresponds to C-O stretch in ester groups, resulting from the attachment of silane groups to the -OH functional groups on the COOH-functionalized SWCNT surface [1, 2].From Fig. S1 (b), it is evident that a covalent Si-O-Si bond exists between Sily-SWCNT and PDMS in the PDMS-SWCNT (Sily) composite, as indicated by the peak at 1010 cm^{-1} [3]. This Si-O-Si bond is also present in PDMS, hence observed in all thin films. However, the intensity of the Si-O-Si bond is higher in the PDMS-SWCNT (Sily) composite due to the presence of the silane functional group on SWCNT. Additionally, in the PDMS-SWCNT (COOH) composite, a weak C=O stretching vibration from the -COOH group is detected at 1640 cm^{-1}, indicating a difference in the chemical bonding between the two types of SWCNT fillers and the PDMS matrix [4]. Notably, this peak is absent in neat PDMS and the PDMS-SWCNT (Sily) composite. The ATR-IR analysis confirms a chemical bonding between Sily-SWCNT and neat PDMS.

S2. Contact Angle Measurement

Contact angle measurements of COOH-SWCNT and Sily-SWCNT coated on glass slides were measured to ensure and quantify the respective hydrophilic and hydrophobic surface polarities of the functionalized carbon nanotubes. The liquid used to study the contact angle of functionalized carbon nanotubes coated on glass slides was distilled water. The measurement was conducted at 25°C under ambient conditions. Three samples of each kind (glass slide, COOH-SWCNT coated glass slides, and sily-SWCNT coated glass slides) were prepared and each sample was tested thrice to produce nine data sets for the measurement of contact angle. The average of the obtained results is discussed in the following section.

Fig. S2 (a) is a plot of the contact angle over time represented for 5 s. The black curve shows the reference plot for the contact angle of distilled water on a clean glass substrate (control sample). The red and blue curves respectively depict the contact angle of sily-SWCNT and COOH-SWCNT coated on glass slides. Fig. S2 (b), (c), and (d) are the contact angle pictures captured at t = 0 s for the glass slide, COOH-SWCNT coated glass slide, and Sily-SWCNT coated glass slide respectively.

The control sample, a glass slide, forms an initial contact angle of 51.4° at t= 0s (Fig. S2 (b)). The cleaned glass slide is slightly hydrophilic promoting the wetting of the glass slide and spreading the water droplet on its surface. The contact angle reduces and stabilizes to 45° as shown in Fig. S2 (a) (Black curve). In the case of a COOH-SWCNT coated glass slide, the water droplet forms an initial contact angle of 18.4° at t= 0 s (Fig. S2 (c)). COOH-SWCNT tends to be highly hydrophilic promoting the wetting of the surface and the spreading of the water droplet to a large surface area within 1 s dropping the water contact angle to 0° instantly (Blue

curve in Fig. S2 (a)). This indicates the highly hydrophilic nature of the COOH-functionalized SWCNT.

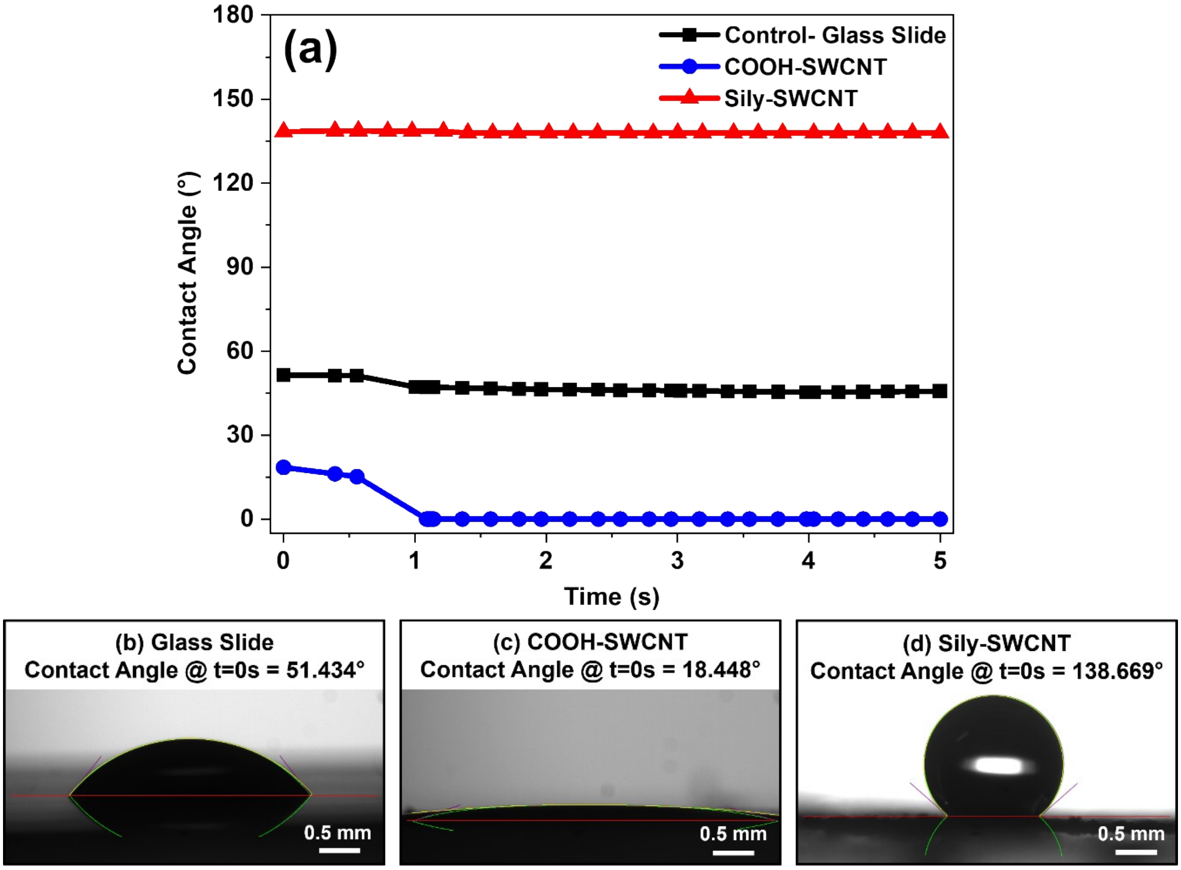

Figure S2. (a) Graphical representation of the change of contact angle of water droplet on the functionalized SWCNT over a time (t) of 5s; Photographic images of the initial contact angle of water droplet at t = 0s for (b) Control sample- Glass Slide, (c) COOH-SWCNT coated glass slide, and (d) Sily-SWCNT coated glass slide.

In the case of the Sily-SWCNT coated glass slide, the water droplet forms an initial contact angle of 138° at t= 0 s (Fig. S2 (d)). Sily-SWCNT tends to be hydrophobic resisting the wetting of the surface and prohibiting the spreading of the water droplet. The water droplet continued to be stable with a contact angle of 138° for over 30 s (Red curve in Fig. S2 (a)). The silane functionalization of SWCNT has produced a hydrophobic organic layer around SWCNT making the surface polarity of the nanotube to be highly hydrophobic. The hydrophobic surface polarity of sily-SWCNT has promoted the interfacial compatibility and dispersion with the hydrophobic PDMS matrix in addition to the chemical adhesion between the silane groups on SWCNT and siloxane groups in PDMS through a Si-O-Si bond. The improvement in chemical and interfacial adhesion of Sily-SWCNT in PDMS has resulted in a homogeneous dispersion promoting the surface mechanics of the composite even at a resolution of 1 µm.

REFERENCES

1. A. Hamed Mashhadzadeh, A. Fereidoon and M. Ghorbanzadeh Ahangari, *Applied Surface Science*, 2017, **420**, 167-179.

2. S. Yellampalli, *Carbon Nanotubes: Polymer Nanocomposites*, BoD–Books on Demand, 2011.
3. D. Bodas and C. Khan-Malek, *Microelectronic engineering*, 2006, **83**, 1277-1279.
4. S. Shin, J. Jang, S.-H. Yoon and I. Mochida, *Carbon*, 1997, **35**, 1739-1743.
